# Inhibitory Effect and Mechanism of Carvacrol against Black Mold Disease Agent *Alternaria alternata* in Goji Berries

**DOI:** 10.3390/jof10060402

**Published:** 2024-06-03

**Authors:** Junjie Wang, Yueli Zhou, Peng Wang, Lunaike Zhao, Huaiyu Zhang, Huan Qu, Fei Xu

**Affiliations:** 1 Key Laboratory of Storage and Processing of Plant Agro-Products, School of Biological Science and Engineering, North Minzu University, Yinchuan 750021, China; 15732950990@163.com (Y.Z.); peng_w08@163.com (P.W.); zhao921526641@163.com (L.Z.); rosalie42@163.com (H.Q.); lengyue0524@163.com (F.X.); 2 College of Life Science, Northwest A & F University, Yangling 712100, China; 3 Physical and Chemical Laboratory of Ningxia Center for Disease Control and Prevention, Yinchuan 750021, China

**Keywords:** carvacrol, *Alternaria alternata*, goji berry, mycotoxin, transcriptomic analysis

## Abstract

*Alternaria alternata*, as a main decay fungus of goji berry, can produce mycotoxins such as alternariol (AOH), alternariol monomethyl ether (AME), and tenuazonic acid (TeA). Carvacrol (CVR) has exhibited a broad-spectrum antifungal activity in vitro. We assumed that CVR can also be applied to control *Alternaria* rot on goji berries and mycotoxins produced by the pathogens. To investigate whether CVR impacts the accumulation of mycotoxins and cell membrane damage of *A. alternata*, the antifungal activity of CVR on the fungal growth and mycotoxin production was evaluated in this study. The results showed that the minimum inhibitory concentration (MIC) of CVR against *A. alternata* was 0.12 µL/mL. Meanwhile, the destruction of plasma membrane integrity, cytoplasmic leakage, intracellular oxidative damage, and inhibitory effect in vivo were also observed in *A. alternata* treated with CVR. Moreover, CVR significantly reduced the accumulation of AOH, AME, and TeA. Transcriptomic profiling was performed by means of comparative RNA-Seq analysis to research the gene expression level of *A. alternata*, which attested to significant changes in nitrogen metabolism, carbon utilization, fatty acid oxidation, and antioxidant enzymes in CVR-treated *A. alternata*. This study suggests a new understanding of the molecular mechanism of response to CVR treatment in *A. alternata*, indicating that CVR is a novel antifungal agent with the potential to be applied to various fungi.

## 1. Introduction

*Alternaria alternata*, a filamentous pathogenic fungus, is widely distributed in soil, air, and crop residues and can be parasitic, saprophytic, and facultative parasitic. The pathogenic fungus causes postharvest rot on many types of fruits such as jujube [1], grape [2], tomato [3], peach [4], mango [5], and sweet cherry [6], resulting in significant economic losses to the fruit industry. *A. alternata* is also one of the primary fungal pathogens which cause the postharvest black mold of goji berries [7]. Meanwhile, the pathogen can produce mycotoxins, including alternariol (AOH), alternariol monomethyl ether (AME), and tenuazonic acid (TeA), which can be accumulated greatly in infected fruits and their processed food products, resulting in a potential food safety hazard and human health issues [8,9,10]. At present, the application of fungicides, including tebuconazole, iprodione, and mancozeb, is considered a primary strategy to control Alternaria rot [11,12,13]. However, it has been reported that the fungicide resistance of pathogens is remarkably raised, posing threats to human health and environmental protection [14]. Therefore, searching for novel approaches is urgently needed to control the postharvest black mold of goji berries.

Essential oils (EOs) and plant-derived antimicrobial agents, which have been used as preservatives or antiseptics and disinfectants, may be natural alternatives to chemical preservatives [15,16,17]. Most importantly, abundant studies have shown that most plant essential oils are generally recognized as safe (GRAS) [18]. The components of EOs and plant-derived antimicrobials, such as carvacrol (CVR) [19], citral [20], magnolol [21], ursolic acid [22], ethyl *p*-coumarate [13,23], and caffeic and *p*- coumaric acids [10], exhibit strong antifungal activity on *A. alternata*. Among them, CVR derived from *Origanum vulgare* L. has attracted more and more attention due to its outstanding biological activities such as antifungal, antioxidant, anticancer, anti–inflammatory, and fresh-keeping activity [24,25]. CVR has been approved by the US Food and Drug Administration (FDA) as a food additive [7]. Research reports show that CVR has obvious broad-spectrum inhibitory effects against various plant pathogenic fungi, including *Aspergillus flavus* [19], *Botrytis cinerea* [19], *Colletotrichum fructicola* [26], *Penicillium digitatum*, and *P. italicum* [27]. However, there are few research reports on CVR’s inhibition of *A. alternata* in goji berries.

High-throughput RNA sequencing analysis has been used to detect mRNA expression profiles of fungal cells that were treated with essential oils, in order to study antifungal mechanisms. Cinnamaldehyde and citral-treated *Penicillium expansum* showed a downregulation of amino and nucleotide sugar metabolism, ergosterol biosynthesis, energy, and amino acid metabolism, along with ATP-binding cassette transporter genes [28]. Li et al.’s [29] study provided insight into the molecular mechanism of tea tree oils against *Botrytis cinerea* and explained the synergistic effect of terpinen–4–ol and 1,8–cineole on *B. cinerea*. Citral-treated *A. alternata* showed a downregulation of genes that affect carbohydrates and amino acids, sulfate and glutathione metabolism, the metabolism of xenobiotics, transporter activity, and mycotoxin biosynthesis [20]. In the magnolol treatment of *A. alternata*, genes that affect global regulators of carbon and nitrogen utilization (such as *CreA* and *NmrA*), the mitogen-activated protein kinase (MAPK) signaling pathway, and mycotoxin biosynthesis were also found to be downregulated [21]. As per our two previous studies, transcriptome analysis showed that a low concentration of CVR (0.06 μL/mL) had an impact on cell wall-related genes in *A. alternata* [8], and the integration analysis of transcriptomics and proteomics further suggested that a low concentration of CVR inhibited *A. alternata* in vitro by interfering with carbohydrate metabolism [30]. This, in turn, affected the growth of the newly forming mycelium’s cell wall, which plays a critical role in the differentiation of the infection structure. However, further research is needed to investigate the transcriptional response of *A. alternata* to higher concentrations of CVR treatment and its effect on toxin accumulation and antifungal activity in goji berry fruit.

Therefore, the aim of the present study is to investigate the antifungal effect of different concentrations of CVR on *A. alternata* in vitro and in vivo, to observe the change in the cell membrane of *A. alternata* treated with CVR, and to determine the effects of CVR on gene expression related to oxidative damage and mycotoxin accumulation in *A. alternata*.

## 2. Materials and Methods

### 2.1. Chemicals, Strains, and Fruit

Carvacrol (CAS: 499-75-2) was obtained from Tokyo Kasei Kogy Co., Ltd. (Shanghai, China) with a purity of 98.0% and 1% (*v*/*v*) Tween-80 was used as a diluent. Analytical standards of alternariol (AOH, CAS: 641-38-3), alternariol monomethyl ether (AME, CAS: 26984-49-5), and tenuazonic acid (TeA, CAS: 610-88-8) were purchased from Romer Labs (Tulln, Austria), and the purities of all the standards were higher than 98%.

*A. alternata* (LTS7-1802) was obtained from the Key Laboratory of Storage and Processing of Plant Agro-Products at North Minzu University in Ningxia, China [7]. The spores were collected from six-day-old potato dextrose agar (PDA) cultures and suspended in 10 mL of sterile distilled water. The number of spores (5 × 10^5^ spores per mL) was determined using a hemocytometer for in vivo and in vitro testing.

Goji berries (*Lycium barbarum* L. cv. Ningnongqi-7) were hand-picked from the National Goji Germplasm Repository, Ningxia Academy of Agricultural and Forestry Sciences, located in Yinchuan, Ningxia, China, on 21 September of 2020. The fruits were packed and stored in a cold storage at 4 °C at the laboratory. Goji berries were used for in vivo assessments within a week.

### 2.2. Antifungal Activity

#### 2.2.1. Mycelial Growth Assays

The effects of CVR on the mycelial growth of *A. alternata* were assessed on PDA plates according to a previously reported method [31]. A 6 mm fungal cake of *A. alternata* was cut with a hole punch from the edge area of the pathogen colony after 6 days of cultivation on PDA plates and placed in the center of the new plates. Autoclaved PDA medium was cooled to about 60 °C, 0.1% streptomycin sulfate was added, and the mixture was poured into Petri dishes (90 mm diameter). The amount of CVR was calculated according to the space of the Petri dish (70 cm^3^), a sterilized filter paper disc (70 mm diameter) was attached on the center of the lid inner surface with different concentrations of CVR (0.03, 0.06, 0.12, and 0.24 μL/mL; the unit was defined as CVR volume per the Petri dish space) added onto the paper, and then the plate was quickly covered. Filter paper without CVR was used as the control. After the plates were incubated at 28 ± 1 °C for 2, 4, and 6 days, the diameter (mm) of the colony area was determined. Each treatment comprised three replicates. The lowest concentration in which no visual hyphal growth was observed after 2 days of incubation was the minimum inhibitory concentration (MIC).

#### 2.2.2. Monitoring Spore Germination and Germ Tube Length

To evaluate the effects of CVR fumigation on the spore germination and tube length of *A. alternata*, the 10 μL spore suspension (5 × 10^5^ spores per mL) was dripped onto the slide (1.5% agar medium) and placed in a large Petri dish with wet filter paper (95% humidity). The amount of CVR was calculated according to the space of the Petri dish (85 cm^3^), a sterilized filter paper disc (70 mm diameter) was attached on the center of the lid inner surface with different concentrations of CVR (0.03, 0.06, 0.12, and 24 μL/mL; the unit was defined as CVR volume per Petri dish space) added onto the paper, and then the plate was quickly covered. Treatment without adding CVR was the control. After 2, 4, 6, and 8 h of incubation, the spore germination rate and tube length were observed and calculated with lactophenol cotton blue staining for 2 min. Each treatment comprised three replicates.

### 2.3. Ultrastructural Observation

*A. alternata* hyphae were cultured on PDA for 3 days and then fumigated with 0.06, 0.12, and 0.24 μL/mL CVR for 48 h, and observed by a JEOL JSM-6360LV scanning electron microscopy (SEM, JEOL, Tokyo, Japan) and an HT7650 transmission electron microscopy (TEM, JEOL, Tokyo, Japan). The hyphae grown without CVR treatment were used as a control. The procedures for the SEM observation were described in a previous study [32].

### 2.4. Determination of Membrane Integrity

The assay for the plasma membrane integrity of the *A. alternata* with different CVR treatments in PDA described above was analyzed using propidium iodide (PI) staining coupled with an DM3000 fluorescence microscope (Leica, Beijing, China) [33].

### 2.5. Cytoplasmic Leakage Assays

The leakage of cytoplasmic contents from *A. alternata* mycelia was measured according to a previously described method with minor modifications [31,34]. A spore suspension of 500 μL (5 × 10^5^ spores per mL) was inoculated in 100 mL potato dextro broth (PDB) medium and cultured at 28 °C and 160 rpm for 2 days. The hyphae were collected by means of centrifugation at 8000× *g* for 10 min, and washed with sterile deionized water 3 times. A total of 1 g of mycelia was resuspended in 20 mL sterile distilled water containing 0.00, 0.06, 0.12, and 0.24 μL/mL CVR, and incubated on a rotary shaker at 28 °C for 0, 30, 60, 90, and 120 min. Cell membrane permeability was measured using an electric conductivity meter (DDS-11A, Shanghai, China). Nucleic acids levels were measured with a microspectrophotometer (Nano-500, Guangzhou, China) at 260 nm (OD260). All experiments were performed in triplicate.

### 2.6. Observation of ROS

*A. alternata* mycelia treated with or without CVR were stained with 10 μM 2,7′-dichlorofluorescein diacetate (DCFH-DA) for half an hour at 37 °C in the dark. After washing 3 times with PBS, the ROS distribution and fluorescence intensity were assessed using a Leica DM3000 microscope (Leica Microsystems CMS GmbH, Wetzlar, Germany) [35].

### 2.7. Determination of Oxidation Resistance

The superoxide dismutase (SOD), catalase (CAT), ascorbate peroxidase (APX), and lipoxygenase (LOX) activities of mycelia were measured using a SOD kit (SOD-1-Y), CAT kit (CAT-1-Y), APX kit (APX-1-W), and LOX kit (LOX-1-W) from Suzhou Comin Biotechnology Co., Ltd. (Suzhou, China), respectively. The content of malondialdehyde (MDA) was measured using the MDA kit (A003-1) of Nanjing Jiancheng Bioengineering Institute (Nanjing, China). The content of hydrogen peroxide (H_2_O_2_) was measured using the Beyotime Biotechnology Co., Ltd. H_2_O_2_ kit (S0038, Shanghai, China).

### 2.8. Mycotoxin Extraction and HPLC-MS Analysis

For mycotoxin extraction, the fungi were cultured in PDA at 28 °C for 3 days and fumigated with CVR (0.06, 0.12, and 0.24 μL/mL), followed by liquid nitrogen grinding of accurately weighed 0.5 g of *A. alternata* mycelium. Subsequently, the hyphae were transferred to 50 mL sterile centrifuge tubes, and 10 mL of acetonitrile, methanol, and 0.05 mol/L pH 3.0 disodium hydrogen phosphate buffer (9:2:9, *v*:*v*:*v*) was added. The solution was thoroughly mixed, received ultrasonic treatment for 15 min, and extracted by shaking at room temperature for 1 h, followed by centrifuging at 9000 r/min at 4 °C for 5 min. A total of 5 mL of supernatant was transferred to a 50 mL sterile centrifuge tube, and 15 mL disodium hydrogen phosphate buffer was added for purification. The HLB purification column (6 cc/500 mg, 60 μm, Waters, Milford, USA) was activated with 5 mL of methanol, ultrapure water, and sodium dihydrogen phosphate buffer, respectively. The solution was purified and eluted with 5 mL methanol. The solution was dried using a nitrogen-blowing instrument at 50 °C, dissolved using 1 mL 10% methanol solution, and eddied at 30 s, followed by centrifuging at 26,000 r/min at 4 °C for 2 min. A total of 200 μL of supernatant was removed to put into a 1.5 mL vial for HPLC analysis.

Separation and a qualitative analysis of AOH, TeA, and AME were performed using an ultra high-performance liquid chromatography tandem mass spectrometer (Lc-20Ad-4000QTRAP, AB Sciex, Framingham, MA, USA). The HPLC conditions were as follows: column, Atlantis T3 (100 mm × 2.1 m × 3 μm); column temperature, 40 °C; and injection volume, 10 μL. Mobile phase A was ammonium acetate aqueous solution, and mobile phase B was methanol. The gradient elution conditions were 10% B retention for 0.0~0.5 min, 10~60% B retention for 0.5~3.0 min, 60~80% B retention for 3.0~5.0 min, 80~100% B retention for 5.0~6.0 min, 100% B retention for 6.0~7.0 min, 100~10% B retention for 7.0~8.0 min, and 10% B retention for 8.0~10.0 min. The mass spectrometry conditions were as follows: ion source mode, anion ion mode (ESI-); mass spectrometry scanning mode, multiple reaction monitoring (MRM); ion source temperature, 550 °C; nebulizer pressure, 40 psi; sheath gas flow, 0.3 mL/min; capillary voltage, −4500 V; other parameters were adjusted to optimum by the instrument. The mass spectrometric parameters such as monitoring ion, cone voltage, and collision voltage of three *Alternaria* species are shown in Table S1.

### 2.9. Transcriptomic Analysis

#### 2.9.1. Total RNA Isolation, cDNA Library Construction, and Sequencing

*A. alternata* was cultured on PDA for 3 days; it was fumigated with 0 and 0.24 μL/mL CVR for 48 h, and the mycelia were collected. Total RNA was extracted from *A. alternata* mycelia using Trizol Reagent Kit (Invitrogen, Carlsbad, CA, USA). RNA quality and purity were monitored by means of 1% agarose gel electrophoresis and Agilent 2100 Bioanalyzer using an RNA 6000 Nano Kit (Agilent Technologies, Santa Clara, CA, USA). Briefly, mRNA was purified from total RNA using poly-T oligo-attached magnetic beads. mRNA was randomly broken by divalent cation in NEB fragmentation buffer, and the database was built according to the common database construction method of NEB. Finally, the library preparations were sequenced on an Illumina Novaseq PE 150 platform and 150 bp paired-end reads were generated. All sequence data were deposited in the NCBI Sequence Read Archive (SRA) under the accession number of the Bioproject PRJNA924017. The accession numbers are SRR26155732, SRR26155733, SRR26155734, SRR26155735, SRR26155736, and SRR26155737, respectively.

#### 2.9.2. Data Analysis, DEGs Screening, and Bioinformatics Analysis

The reads adapters, low quality reads, reads with more than 5% N (N: ambiguous bases information), and over-short sequences of the length were to be trimmed from the raw reads using Trim-galore software. The remaining reads were subjected to bioinformatics analysis based on the genome annotation of *A. alternata* FERA 1177 (GCA_004154755.1) (https://www.ncbi.nlm.nih.gov/genome/11201?genome_assembly_id=448691, accessed on 18 October 2021) using the software of Hisat 2.0 [36]. The FPKM of each gene was calculated based on the length of the gene and read count mapped to this gene. A differential expression analysis of two conditions was performed using the Cufflinks-2.2.1. The *p* values were adjusted using the Benjamini and Hochberg method. A corrected *p*-value of 0.05 and an absolute foldchange of 2 were set as the thresholds for significantly differential expression. Finally, gene ontology (GO) functional annotation and Kyoto encyclopedia of genes and genomes (KEGG) pathway enrichment analysis were carried out to understand the functions of the differentially expressed genes (DEGs).

#### 2.9.3. Gene Expression Using Real-Time RT-PCR Analysis

Total RNA was extracted using Trizol Reagent Kit (Invitrogen, Carlsbad, CA, USA), and cDNA was synthesized using a TransScript^®^ one-step gDNA removal and cDNA synthesis supermix kit (Transgen). Gene primers were designed using Primer Premier 5.0 software (Premier Biosoft, Burlington, Canada, Table S2). *β*–tubulin was selected as the reference gene from *A. alternata*. The quantitative real-time PCR (qPCR) amplification reactions were performed using a Roche Light Cycler 480II (Roche, Mannheim, Germany). The reaction mixture contained 10 μL of SYBR Green QPCR, 2.0 μL of cDNA, 0.8 μL of primers (10 μM), and 6.4 μL of ddH_2_O. The qPCR reaction parameters were 95 °C for 10 s, followed by 40 cycles of 95 °C for 10 s, and 60 °C for 30 s. The relative gene expression was calculated using the 2^−ΔΔCT^ method [37]. Three replicate measurements were performed on each sample.

### 2.10. Effect of CVR on A. alternata in Goji Berry

The in vivo assays were carried out according to a previously described method with subtle modifications [34]. Goji berries were surface-disinfected using 75% alcohol and wounded on the epidermis in the equatorial region with a sterile punch (1 mm deep and 1 mm wide). Each wound site was inoculated with 2 μL of spore suspension at 5 × 10^5^ spores per mL *A. alternata*, and then air-dried at room temperature. Selected goji berries were placed in sealed boxes and fumigated with CVR (0.00, 0.06, 0.12, and 0.24 μL/mL) for 24 h, balanced for 6 h, and stored at room temperature (28 ± 2 °C), 90 ± 3% relative humidity for 2 days, and then the lesion diameter was measured (mm). Each treatment contained ten fruits. The experiment was repeated three times.

### 2.11. Statistical Analysis

All experiments were conducted in duplicate. The results were analyzed using GraphPad Prism 5.0 software (GraphPad Inc., La Jolla, CA, USA) and one-way analysis of variance (SPSS 23.0; IBM Corp., Armonk, NY, USA). Mean differences were compared using Student’s *T*-tests, with a significant level of *p* < 0.05.

## 3. Results

### 3.1. CVR Inhibits the Colony Growth, Spore Germination, and Germ Tube Length of A. alternata

**Figure 1:**
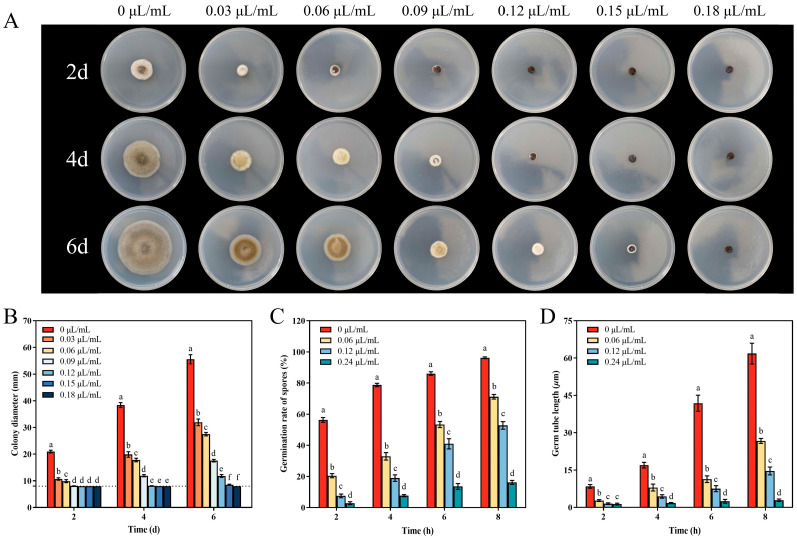
Effect of CVR on colony morphology (**A**), colony diameter (**B**), spore germination rate (**C**), and germ tube length (**D**) of *A. alternata*. Colony diameter was examined every 2 days after incubation at 28 °C for a total of 6 days. According to Duncan’s multiple comparisons, different letters indicate significant differences (*p* < 0.05).

Figure 1A,B show that CVR can inhibit the growth of *A. alternata* colonies in a concentration-dependent manner. After 6 days of treatment, the diameter of colonies treated with CVR at concentrations of 0.03, 0.06, 0.09, 0.12, 0.15, and 0.18 μL/mL were reduced by 49.76%, 59.06%, 79.95%, 91.99%, 98.97%, and 100%, respectively, when compared to the control group at the same time (Figure 1B). The results also showed that CVR treatment completely restrained the mycelial growth of *A. alternata* at the concentration of 0.12 μL/mL after 2 days, while CVR at the concentration of 0.15 μL/mL completely inhibited mycelial growth on the 4th day (Figure 1A,B). As a result, the MIC and MFC of CVR against *A. alternata* were evaluated as 0.12 μL/mL and 0.15 μL/mL, respectively.

The effect of different concentrations of CVR on the spore germination in *A. alternara* is shown in Figure 1C. When *A. alternata* was exposed to a concentration of 0.12 μL/mL of CVR for 8 days, there was a 45.15% reduction in spore germination compared to the control at the same time. In addition, when *A. alternata* was fumigated with CVR at a concentration of 0.24 μL/mL for 8 days, spore germination was completely inhibited (Figure 1C). Similarly, germ tube length was markedly inhibited in response to different concentrations of CVR during various stages of cultivation (Figure 1D). After being fumigated with CVR at a concentration of 0.12 μL/mL for 8 days, the germ tube length of *A. alternata* was found to be 76.32% shorter compared to the control at the same period. When the concentration was increased to 0.24 μL/mL, hardly any growth was observed (Figure 1D).

### 3.2. Morphological and Ultrastructural Alterations of A. alternata

**Figure 2:**
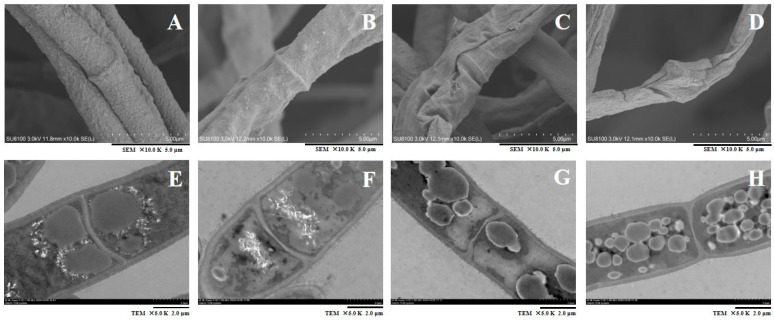
Microstructure observation of the effect of CVR on the mycelium morphology of *A. alternata*. (**A**–**D**) SEM images of treated 0.00, 0.06, 0.12, and 0.24 μL/mL CVR, respectively (×10,000). Scale bars: 5.0 μm. (**E**–**H**) TEM length cutting images of treated 0.00, 0.06, 0.12, and 0.24 μL/mL CVR, respectively. (**E**–**H**) magnification is ×5000 and scale bars are 2.0 μm.

SEM analysis revealed that the control samples had a normal structure and consistent, uniform, and strong hyphae of a fixed width (Figure 2A). However, the hyphae treated with CVR showed significant changes in their morphology. The hyphae appeared as small, dot-like particles on the surface, with distorted, partially flattened, and withered hyphae (Figure 2B–D). Additionally, the extent of damage to the hyphae increased with the concentration of CVR.

The effects of CVR on the internal structure of *A. alternata* hyphae cells were observed using TEM. The results are presented in Figure 2E,H. The hyphae that were not treated with CVR had a complete cell wall, smooth cell membranes, and a compact cell structure with homogeneous cytoplasm. However, after being treated with CVR, the structure of the cytoplasm of the hyphae solidified, resulting in the separation of the cytoplasm wall and the fusion of organelles. As the concentration of CVR increased, the damage to the cell wall and the internal structure of the cell membrane of hyphae became more severe, and the organelle fusion also became more pronounced.

### 3.3. Alterations to the Cell Membrane Integrity of A. alternata following CVR Treatment

**Figure 3:**
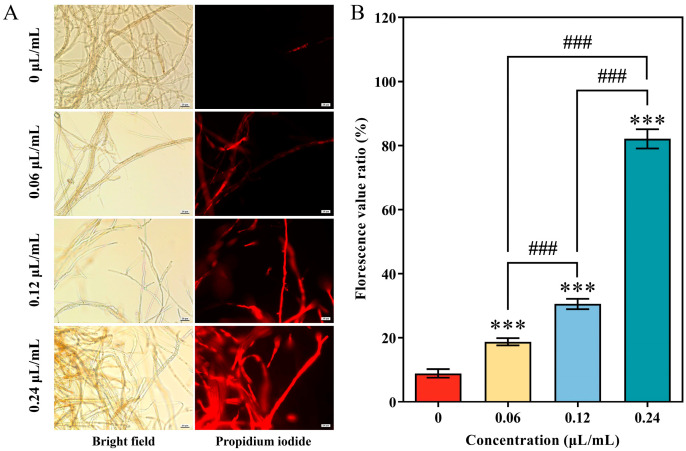
Plasma membrane integrity of *A. alternata* mycelia treated with CVR at various concentrations. (**A**) Propidium iodide (PI) staining assay. Scale bars: 20 μm. (**B**) Florescence value ratio. Red fluorescence indicates mycelia with disrupted plasma membranes. Bars represent 20 μm. *** (*p* < 0.001) vs. control group, ### (*p* < 0.001) between different treatments.

The mycelial cells were stained with PI in the presence and absence of CVR. The results indicated that the cell membrane integrity of *A. alternata* decreased in a concentration-dependent manner with CVR treatment (Figure 3A). This was evident from the increased staining intensity observed in PI staining (Figure 3B). At a concentration of 0.24 μL/mL of CVR, 81% of the cells showed impaired membrane integrity with PI fluorescence.

### 3.4. Damages of the Permeability of Plasma Membrane of A. alternata after CVR Treatment

**Figure 4:**
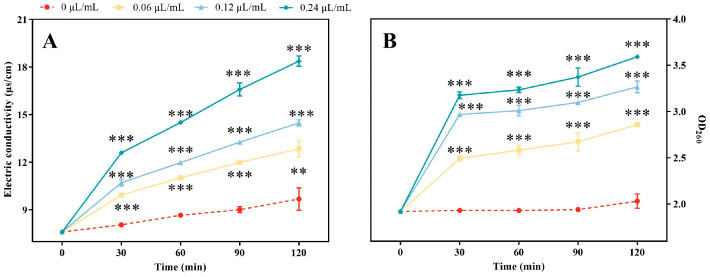
Effect of CVR on electrical conductivity (**A**) and nucleic acid leakage (**B**) of *A. alternata*. Vertical bars represent standard errors. *** (*p* < 0.001) vs. control group.

To evaluate the plasma membrane permeability of *A. alternata*, electrolyte leakage and nucleic acid content were measured. The results showed that the application of CVR increased the electrolyte leakage in *A. alternata*. After 120 min, the electrical conductivity of *A. alternata* cells treated with 0.06, 0.12, and 0.24 μL/mL CVR was significantly higher by 12.84, 14.48, and 18.37 μs/cm, respectively, in comparison to the control (Figure 4A). Moreover, the treatment of *A. alternata* with CVR also resulted in electrolyte leakage after 30 min and increased nucleic acid content. After 120 min of CVR treatment, the OD_260_ in the CVR-treated *A. alternata* with concentrations of 0.06, 0.12, and 0.24 μL/mL were 2.86, 3.27, and 3.59 times higher than the control, respectively (Figure 4B).

### 3.5. Distribution of Endogenous ROS in A. alternata Mycelia after CVR Treatment

**Figure 5:**
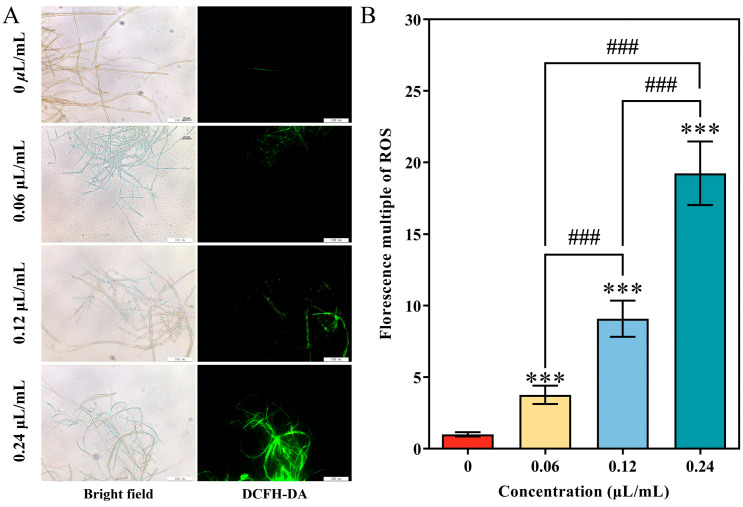
Effects of CVR treatment on fluorescent microscope images (**A**) and fluorescent intensity (**B**) of endogenous ROS in *A. alternata* mycelia. *** (*p* < 0.001) vs. control group, ### (*p* < 0.001) between different treatments.

The distribution of endogenous ROS in *A. alternata* was observed using DCFH-DA with fluorescence microscope. As shown in Figure 5A, compared with the unexposed control, CVR exposure led to a significantly visible green fluorescence, indicating the accumulation of endogenous ROS in *A. alternata* mycelia with CVR treatment (Figure 5A). The effects of CVR on the accumulation of ROS was further evident from the florescence multiple of ROS, as 0.06, 0.12, and 0.24 μL/mL CVR treatment significantly increased the fluorescence intensity related to ROS accumulation in the mycelia to 3.76-fold, 9.09-fold, and 19.31-fold that in the control group, respectively (Figure 5B).

### 3.6. Disarrangement of Redox Homeostasis of A. alternata after CVR Treatment

**Figure 6:**
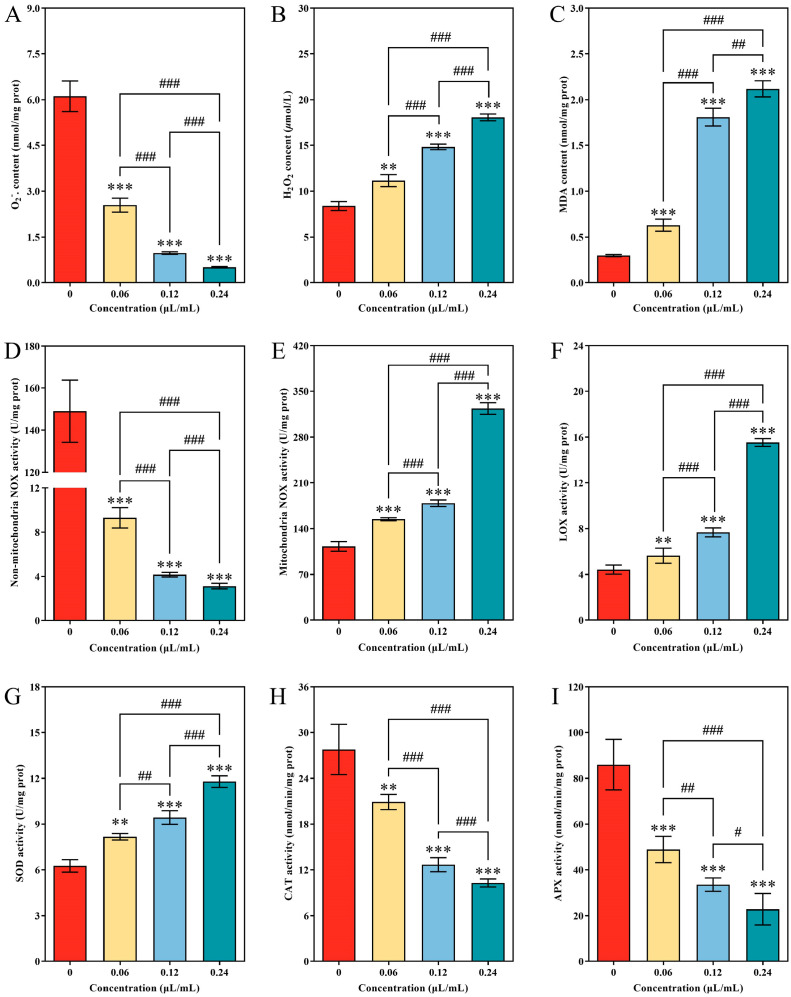
Effects of CVR on antioxidant substances and antioxidant enzyme activity of *A. alternata*. (**A**) O_2_·^−^, (**B**) H_2_O_2_, (**C**) MDA, (**D**–**F**) LOX, (**G**) SOD, (**H**) CAT, and (**I**) APX. ** (*p* < 0.01), *** (*p* < 0.001) vs. control group, # (*p* < 0.05), ## (*p* < 0.01), ### (*p* < 0.001) between different treatments.

The effect of CVR treatment on the content of O_2_·^−^ and H_2_O_2_ of *A. alternata* is shown in Figure 6A,B. CVR treatment significantly reduced O_2_·^−^ content and enhanced the accumulation of H_2_O_2_. These different effects of CVR on two types of reactive oxygen species become more significant with increasing concentrations (Figure 6A,B). The content of O_2_·^−^ in the mycelia exposure to 0.06, 0.12, and 0.24 μL/mL CVR was 58.36%, 84.17%, and 91.73% lower than that of control (Figure 6A), while the H_2_O_2_ content of untreated mycelia was 24.83%, 43.44%, and 53.63% lower than that of 0.06, 0.12, and 0.24 μL/mL CVR treatment, respectively (Figure 6B). Exposure to CVR in *A. alternata* resulted in an increase in MDA content. The MDA content of mycelia with 0.06, 0.12, and 0.24 μL/mL CVR treatment was 53.19%, 83.70%, and 86.07% higher than the control, respectively (Figure 6C). CVR treatment activated the activity of three enzymes including mitochondrial NOX, LOX, and SOD. The activity of mitochondrial NOX, LOX, and SOD in 0.24 μL/mL of CVR treatment groups were 186.82%, 250.75%, and 88.43% higher than that of the corresponding control groups, respectively (Figure 6E–G). On the contrary, the other three types of enzyme-related ROS metabolism containing non-mitochondrial NOX, CAT, and APX were inhibited by CVR treatment (Figure 6D,H,I). The activity of non-mitochondrial NOX, CAT, and APX in 0.24 μL/mL of CVR treatment groups was only 2.11%, 37.03%, and 26.56% of the corresponding control groups, respectively.

### 3.7. Repression of Mycotoxins Produced by A. alternata following CVR Treatment

**Figure 7:**
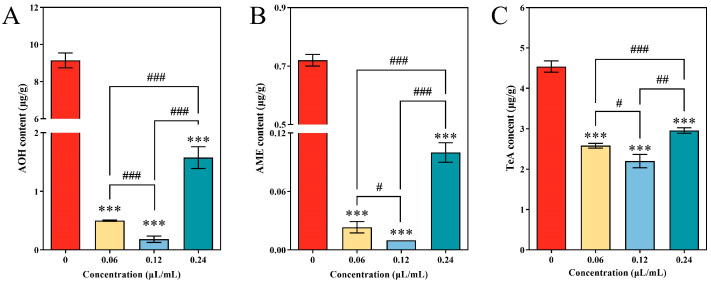
Alternariol (AOH), alternariol monomethyl ether (AME), and tenuazonic acid (TeA) contents of *A. alternata* treated with CVR. The amounts of the metabolites were determined through HPLC using triplicate samples and are represented as means and standard deviations. *** (*p* < 0.001) vs. control group, # (*p* < 0.05), ## (*p* < 0.01), ### (*p* < 0.001) between different treatments.

Three types of mycotoxins, namely, AOH, AME, and TeA, were extracted and detected from the mycelia of *A. alternata* using UPLC-TOF-ESI-MS. The content of AOH, TeA, and AME in *A. alternata* mycelium decreased significantly with the increase in CVR concentration (Figure 7). In the CVR treatment groups with a concentration of 0.12 μL/mL, the content of AOH, AME, and TeA was only 2.00%, 1.39%, and 48.46% of the corresponding control groups, respectively.

### 3.8. Global Analysis of Transcriptomic Profile

A comparison of the transcriptomes of *A. alternata* was carried out to uncover the potential antifungal and anti-mycotoxigenic mechanisms between control and CVR. The total statistics of the RNA-seq data are provided in Table S3. After removing low-quality reads, adaptor sequences, and ambiguous bases among the top 10% of the sequences, over 21 million clean reads were obtained. Based on the alignment with the reference genome sequence, approximately 82.19% of the total mapped reads was averaged. The biological replicates had a higher correlation than 98% based on the expression matrix using Pearson’s correlation coefficient. Using Veen analysis, 12562 and 12421 expressed genes were found in the control and CVR treatment, respectively (Figure S1A,B). It was found that 260 genes were exclusively transcribed in the control group and 119 genes were solely expressed in the CVR group.

RNA-Seq data were studied in-depth to identify genes and pathway differential expression in *A. alternata* when CVR is present. By using fold changes of ≥2 and false discovery rates of <0.05, the analysis revealed differentially expressed genes (DEGs), as shown in Table S4. The results showed that 4311 DEGs were identified in the two groups of fungi treated with CVR and untreated fungi, with 1803 (42%) being upregulated and 2508 (58%) being downregulated when compared to control (Figure S1A,C).

### 3.9. Functional Annotation of DEGs

**Figure 8:**
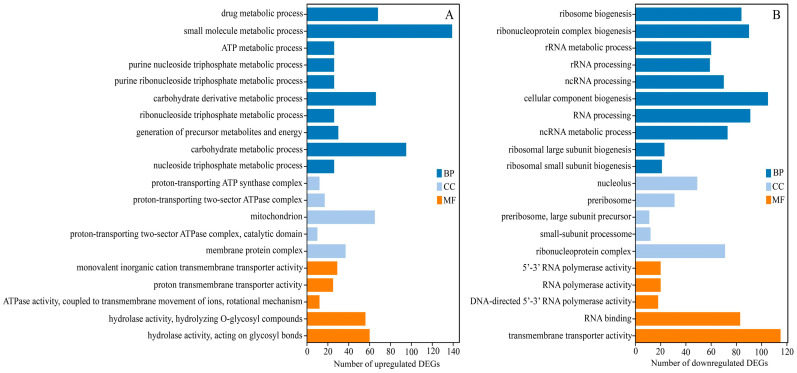
Gene ontology (GO) enrichment analysis of differentially expressed genes (DEGs). Top 20 results of the highest enrichment level were separately obtained from the analysis of upregulated DEGs (**A**) and downregulated DEGs (**B**). The columns of dark blue, light blue, and orange represent biological process (BP), cellular component (CC), and molecular function (MP), respectively.

Through the functional analysis of these DEGs, we explored the molecular-level mechanisms of inhibition. The GO enrichment analysis yielded different significant results for the upregulated and downregulated DEGs (Figure 8A,B). The upregulated DEGs were significantly enriched in primary metabolism and ATP, including small molecule and carbohydrate metabolic processes, mitochondria, and hydrolase activity, which are crucial for survival during stress (Figure 8A). On the other hand, the downregulated DEGs were mostly enriched in biological processes, cellular components, and molecular functions, such as cellular component biogenesis, RNA processing, nucleolus, ribonucleoprotein complex, and ribonucleoprotein complex (Figure 8B).

A KEGG pathway enrichment analysis was performed to determine the biological significance of DEGs between CVR and CK groups. A total of 25 KEGG pathways were enriched in the transcriptome, among which the top 5 KEGG pathways enriched with the most DEGs were biosynthesis of secondary metabolites, biosynthesis of cofactors, carbon metabolism, biosynthesis of amino acids, and oxidative phosphorylation (Figure S2).

### 3.10. Analysis of DEGs

**Figure 9:**
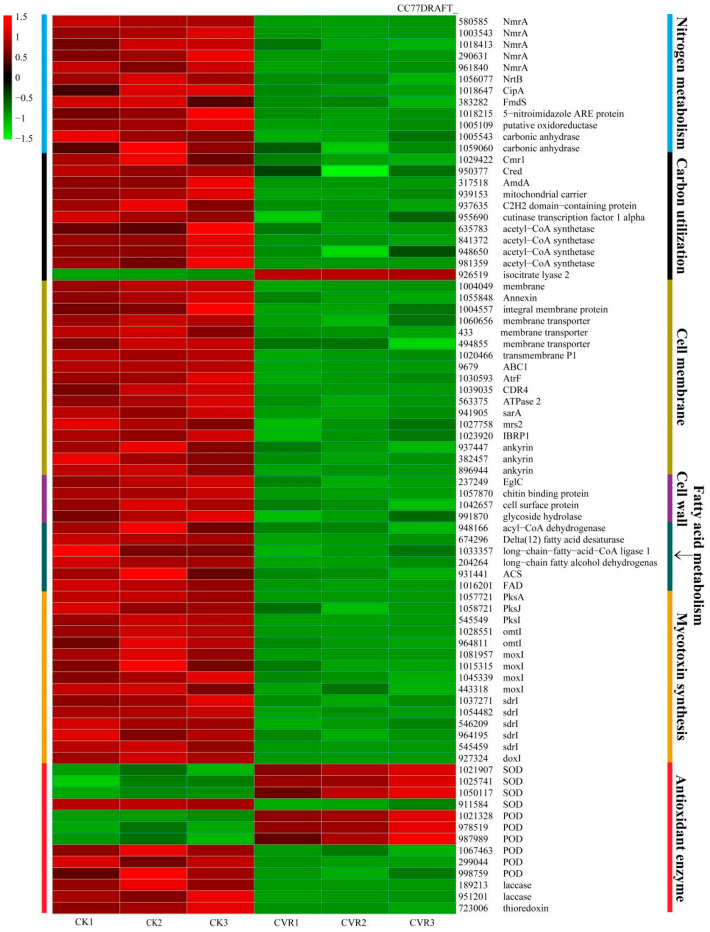
Heatmaps showing relative expression for selected DEGs.

In Figure 9, it is shown that the differential genes were mainly related to seven biological processes such as nitrogen metabolism, carbon utilization, cell membrane, cell wall, fatty acid metabolism, mycotoxin synthesis and antioxidant enzyme. Nitrogen metabolism and carbon utilization are related to the nutritional growth and biosynthesis of *A. alternata*. After the CVR treatment, 12 genes involved in nitrogen metabolism were downregulated, while 1 gene was upregulated and 10 genes were downregulated in carbon utilization. These indicated that the CVR treatment inhibited nitrogen metabolism and carbon utilization, with the exception of isocitrate lyase CC77DRAFT 926519 in *A. alternata*. Following the CVR treatment, there was significant downregulation in 17 genes related to cell membrane transport and 4 genes related to cell wall construction in *A. alternata*. Additionally, the expression of 6 genes related to fat metabolism and 15 genes related to mycotoxin synthesis was inhibited by the CVR treatment. Furthermore, the genes regulating SOD, POD, laccase, and thioredoxin in *A. alternata* showed differential expression after the CVR treatment. Among them, two genes regulating laccase and one gene regulating thioredoxin were downregulated. Notably, the genes regulating SOD and POD exhibited both upregulation and downregulation simultaneously.

### 3.11. qRT-PCR Analyses of Selected DEGs

The results of the transcriptome analysis of the CVR treatment group were validated through qRT-PCR using seven selected genes. As shown in Figure S3A, the expression of *catalase/peroxidase HPI* (*CC77DRAFT_978519*) in the CVR-treated group was upregulated in both the RT-qPCR and RNA-Seq. On the other hand, the expression of *cytosolic Cu/Zu superoxide dismutase* (*CC77DRAFT_911584*), *heme peroxidase* (*CC77DRAFT_299044*), *mitochondrial carrier* (*CC77DRAFT_939153*), *nmr A family protein* (*CC77DRAFT_580585*), *putative oxidoreductase* (*CC77DRAFT_1005109*), and *polyketide synthase PksA* (*CC77DR AFT_1057721*) in the CVR treatment group was found to be downregulated in the RT-qPCR and RNA-Seq (Figure S3B–G). These results are consistent with the findings from RNA-Seq (Figure S3).

### 3.12. Inhibition of the Lesion Development of Black Mold in Goji Berry

**Figure 10:**
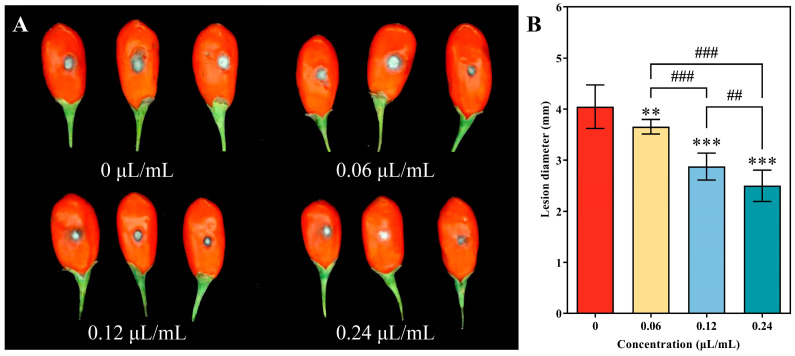
Disease severity of spot disease caused by *A. alternata* in goji berries stored at 28 ± 3 °C and efficacy of CVR at various concentrations. (**A**) Goji berries at 2 days of storage. (**B**) Statistical analysis of lesion diameter shown as histograms. ** (*p* < 0.01), *** (*p* < 0.001) vs. control group, ## (*p* < 0.01), ### (*p* < 0.001) between different treatments.

It has been found that the use of CVR for fumigation is effective in preventing the development of black mold on goji berries which have been inoculated with *A. alternata*. As shown in Figure 10, after two days of inoculation, the lesion diameters of the berries were reduced by 9.63%, 28.89%, and 38.27% with 0.06, 0.12, and 0.24 μL/mL CVR treatment, respectively, compared to the untreated berries (Figure 10B). The highest level of disease control was observed in berries treated with 0.24 μL/mL of CVR (Figure 10A).

## 4. Discussion

The application of essential oils (EOs) for managing postharvest diseases in fruits and vegetables is a popular and growing research area [38,39]. CVR, the main compound in oregano EOs, has strong antifungal potential for controlling postharvest fungal infections in fresh produce, such as blue mold on lemon fruit by *P. italicium* [27], soft rot on peaches by *Rhizopus stolonifer* [40], and anthracnose on red pitaya by *Colletotrichum fructicola* [26]. Although it has been proven that CVR has an antifungal effect against postharvest pathogens, the detailed inhibitory mechanism of CVR is not well understood. In our current study, we discovered that CVR effectively inhibits the mycelial extension, spore germination, and germ tube elongation of *A. alternata*. The strength of inhibition was positively correlated with the concentration of CVR. Moreover, the MIC of CVR on *A. alternata* was significantly lower than that of other natural substances, such as essential oil citral, magnolol, *p*-coumaric acid, caffeic acid, and ferulic acid [10,20,21,23]. Further investigation indicated that CVR also could delay black mold in goji berries.

*A. alternata* is an important plant pathogen, which can produce mycotoxins during the infection process, causing a huge threat to food and feed safety. *Alternaria* mycotoxins including AOH, AME, TeA, tentoxin (TEN), altenuene (ALT), and altertoxins (ATX) are secondary metabolites from *A. alternata*. Of these mycotoxins, two of the most frequent ones are AOH and AME [21,34,41,42,43]. In order to control this contamination, essential oils have been proven to be environmentally friendly alternatives to common antifungal agents, particularly when food and feed have been contaminated by *Alternaria* mycotoxins after harvest. There is some research evidence that volatile compounds affect mycotoxin synthesis, but the effect of volatile essential oil CVR on mycotoxins has been little reported. To the best of our knowledge, this study reported for the first time that CVR has a strong inhibitory effect on the synthesis of AOH and AME (Figure 7). Previous studies have showed that phenolics demonstrated a strong inhibition of trichothecenes biosynthesis by *Fusarium* [44,45], aflatoxin and ochratoxin A production by *Aspergillus* [46,47], and AOH and AME synthesis by *Alternaria* [21], which may be due to the presence of free phenolic hydroxyl groups on phenolic compounds. Wang et al. [10] reported that phenolics may be effective inhibitors for the synthesis of *Alternaria* mycotoxins. On this basis, transcriptome analysis was used to further explore the antifungal and antimycotoxigenic mechanism of CVR on *A. alternata* growth and mycotoxin production.

In *A. alternata*, the production of AOH and its derivatives is controlled by a polyketide synthase (*Pks*) gene cluster, where *aohR* (Gal4-like transcription factor) is used as a positive transcription factor for toxin biosynthesis [48]. In addition, four enzymes including *omtI* (an O-methyl transferase), *moxI* (a mono oxygenase), *sdrI* (a short chain dehydrogenase), and *doxI* (an estradiol dioxygenase) were involved in the biosynthesis of *Alternaria* mycotoxins [48]. In *A. alternata*, *PksI* is necessary for AOH synthesis, whereas *omtI* is required for AME synthesis [48]. Our RNA-Seq data showed that the gene clusters (such as *PksA*, *PksJ*, *PksI*, *omtI*, *moxI*, *sdrI*, and *doxI*) involved in the biosynthesis of AOH and AME were significantly downregulated, but the expression of the regulatory gene *aohR* did not change after CVR treatment (Figure 9). According to the research evidence of Wang et al. [21,22], the expression of *pksI* and *omtI* was significantly downregulated by the essential oils citral and magnolia, but the regulatory factor *aohR* did not significantly change. In the aflatoxin production gene cluster, Zhao et al. [47] found that gallic acid did not significantly affect regulatory factor *aflR* and *aflS*, but most structural genes were prominently downregulated. In summary, these results suggested that CVR may lower mycotoxin biosynthesis by inhibiting *pksA*, *PksJ*, *PksI*, *omtI*, *moxI*, *sdrI*, and *doxI* expression.

The growth and mycotoxin biosynthesis in fungi is also influenced by carbon, nitrogen, and fatty acid metabolism and the corresponding regulators [47,49,50]. Interestingly, nitrogen sources dominate the biosynthesis of AOH and AME more than carbon sources [49], and fatty acid metabolism directly affects the production of both mycotoxins [34]. The transcription factors *NmrA*, *NrtB*, *Cred*, and *Cmr1* play an important role in regulating the expression of carbon and nitrogen source utilization genes [50,51,52]. Han et al. [51] showed that the lack of *NmrA* had an inhibitory effect on the growth of *Aspergillus flavus* and the production of aflatoxin. As a member of the nitrate assimilation system, *NrtB* is essential for the use of non-preferred nitrogen sources [53]. Therefore, the CVR treatment of *A. alternata* led to a significant downregulation of the transcription factors *NmrA* and *NrtB* (Figure 9), which may be related to the inhibition of its growth and *Alternaria* toxin production. In *A. flavus*, the absence of *CreA* caused an almost complete inhibition of aflatoxin biosynthesis [47]. However, the CVR treatment of *A. alternata* led to the downregulation of *Cred*; *Cred* and *CreA* may be members of the same family and have the same biological functions, so the downregulation of *Cred* may also be related to *Alternaria* toxin biosynthesis. Jiang et al. [52] reported that the transcriptional activator *Cmr1* played a key regulatory role in the cell wall integrity (CWI) signaling pathway in the *Aureobasidium melanogenum* XJ5–1. The CWI signaling pathway was essential for cell adaptation to stress, cell wall maintenance, and mycotoxin biosynthesis [54,55]. The transcriptional activator *Cmr1* was downregulated, which may be one of the reasons for the imbalance of cell oxidative stress, cell wall destruction, and decreased toxin content caused by the CVR treatment of *A. alternata*. Furthermore, fatty acid biosynthesis affects the fluidity and rigidity of the membrane [34]. Acetyl-CoA is the precursor for the production of AOH and AME, which is mainly produced by fatty acid *β*-oxidation and pyruvate decarboxylation [47]. Zhao et al. [47] and Wang et al. [34] reported that gallic acid and magnolol inhibit the formation of mycotoxins by reducing the expression of fatty acid *β*-oxidation genes. In summary, after the CVR treatment of *A. alternata*, the expression of genes related to nitrogen metabolism, carbon utilization, and fatty acid metabolism was significantly downregulated, which may be one of the main reasons why CVR inhibits the toxin production and growth of *Alternaria*.

In addition, fungal oxidative damage is also considered to be an important factor in the antifungal and suppressive mycotoxin peculiarities of essential oils [28,56,57]. In response to stress, reactive oxygen species (ROS) are ineluctably produced from plant essential oils, which can lead to cell plasma membrane peroxidation, fatty acid oxidation, DNA oxidation, protein oxidation damage, etc. The overproduction of ROS can cause fungal cell growth inhibition and even death. Furthermore, enzyme systems, such as catalases and superoxide dismutases, play a very important role in the antioxidant defense system of fungi under ROS stress [58]. In this study, the CVR treatment of *A. alternata* resulted in a significant increase in SOD enzyme activity and excessive H_2_O_2_ production, while CAT and APX enzyme activities decreased significantly, and the overaccumulated H_2_O_2_ could not be converted into H_2_O (Figure 6). The increased expression of three genes encoding *SOD* in *A. alternata* after CVR treatment suggested that CVR promoted the production of H_2_O_2_. The thioredoxin system, which consists of thioredoxin proteins and their respective reductases, is the most common and conserved system used to remove ROS [59]. The downregulation of *thioredoxin* indicated that the ability to clear ROS in *A. alternata* was inhibited. During the growth process of fungi, POD has different effects based on location. Class III POD produces ROS in fungal vacuoles, while cytoplasmic POD further converts H_2_O_2_ produced by thioredoxin and SOD into H_2_O [59]. Furthermore, the simultaneous upregulation of two genes and downregulation of two genes of *POD* indicated that CVR treatment affects peroxidase activity in *A. alternata*. The excessive production of H_2_O_2_ leads to an increase in LOX enzyme activity, significantly increases the MDA content of the cell membrane, and destroys the integrity of the cell membrane. The PI and DCFH-DA staining, electrolyte leakage, nucleic acid content, and transmission electron microscopy results of hyphae are consistent with the results of the ROS enzyme system. ROS play an important role in many processes in the fungal cell, such as growth, development, metabolism, and the biosynthesis of secondary metabolites. Therefore, maintaining ROS levels is essential to the fungi’s survival. After CVR treatment, the antioxidation in *A. alternata* was significantly reduced, ROS were accumulated in excess, and the enzyme system of ROS was inhibited and failed to clear overaccumulated ROS in time. Eventually, the growth of *A. alternata* was suppressed. At the same time, the disordered ROS also caused the mycotoxins in the secondary metabolites to be suppressed.

## 5. Conclusions

**Figure 11:**
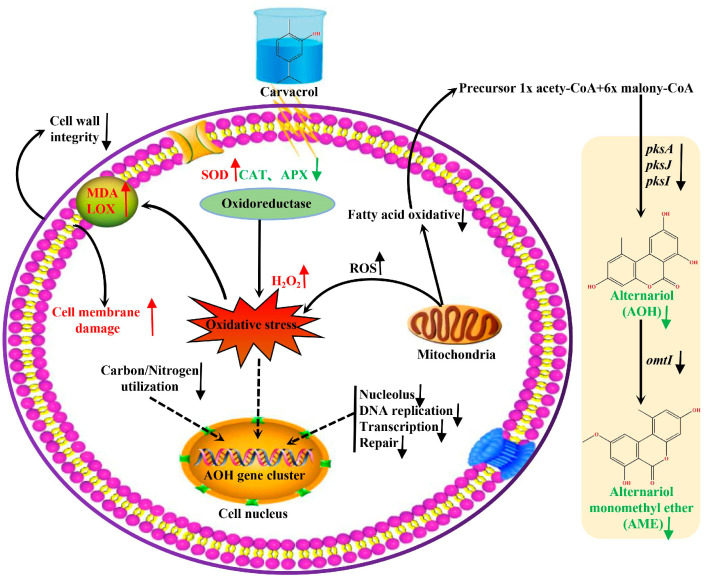
Model diagram showing the antifungal activity of CVR against *A. alternata* by means of mycotoxin production repression and membrane oxidative damage.

This study found that CVR can inhibit the growth of *A. alternata* and the biosynthesis of mycotoxins through cell membrane destruction and ROS disorder. The results of our research are shown in Figure 11: (1) CVR inhibits the biosynthesis of AOH and AME by downregulating polyketide clustered genes, including *pksA*, *pksJ*, *pksI*, and *omtI*; (2) CVR inhibits nitrogen metabolism, carbon utilization, and fatty acid oxidation, so that the growth of *A. alternata* and the biosynthesis of mycotoxins are inhibited; (3) CVR destroys the ROS enzyme system, causing H_2_O_2_ overaccumulation to destroy cell membranes and cell walls; and (4) CVR could also cause a decline in nucleus, DNA replication, transcription, and repair. In summary, considering it could reduce mycotoxin contamination and control *Alternaria* decay, CVR may be an alternative to traditional fungicides.

## Data Availability

Data are contained within the article.

## Supplementary Materials

The following supporting information can be downloaded at: https://www.mdpi.com/article/10.3390/jof10060402/s1, Figure S1: Summary of RNA-Seq analysis; Figure S2: Analysis of the KEGG enrichment of differential expressed genes; Figure S3: Differential gene analysis of RT-qPCR and RNA-Seq; Table S1: Optimized multiple reaction monitoring (MRM) parameters for AOH, TeA, and AME mycotoxins; Table S2: Primers used in RT-qPCR experiment; Table S3: Summary of the RNA-Seq data in the control (CK) and 0.24 μL/mL carvacrol-treated *A. alternata* (CVR); Table S4: Differentially expressed genes (DEGs) between the control and CVR-stressed samples of *A. alternata*.
