# Supplementary material for: Inhibitory Effect and Mechanism of Carvacrol against Black Mold Disease Agent Alternaria alternata in Goji Berries
Source: J Fungi (Basel). 2024 Jun 3;10(6):402. doi: 10.3390/jof10060402 (PMC11204410; doi:10.3390/jof10060402)
Supplement: Supplementary file 1 [file jof-10-00402-s001.zip › Figure S1-S3 and Table S1-S3.pdf]

Antifungal activity of carvacrol against black mold disease agent *Alternaria alternata* on goji berries by mycotoxin production repression and membrane oxidative damage

Junjie Wang [1,*,†], Yueli Zhou [1,†], Peng Wang [1,2,†], Lunaike Zhao [1], Huaiyu Zhang [1,*], Huan Qu [1] and Fei Xu [1,3]

[1] Key Laboratory of Storage and Processing of Plant Agro-Products, School of Biological Science and Engineering, North Minzu University, Yinchuan 750021, China; smkxwjj@163.com (J.W.); 15732950990@163.com (Y.Z.); Peng_W08@163.com (P.W.); zhao921526641@163.co (L.Z.); domybest-001@163.com (H.Z.); rosalie42@163.com (H.Q.); lengyue0524@163.com (F.X.)
[2] College of Life Science, Northwest A & F University, Yangling 712100, China
[3] Physical and Chemical Laboratory of Ningxia Center for Disease Control and Prevention, Yinchuan 750021, China.
* Correspondence: smkxwjj@163.com (J.W.); domybest-001@163.com (H.Z.)
† These authors contributed equally to this work.

Figure. S1 . Summary of RNA-Seq analysis. (A) Statistical scatter diagram of differential gene expression. (B) Venn diagram of the transcribed genes between carvacrol treated and untreated samples. CK, no carvacrol treatment. CVR, 0.24 μl/mL carvacrol treatment. (C) Number of differentially expressd genes (DEGs) in *A.alternata* exposed to carvacrol stress. Out of 4311 DEGs, 1803 genes were highly up-regulated, and 2508 genes were markedly down-regulated.

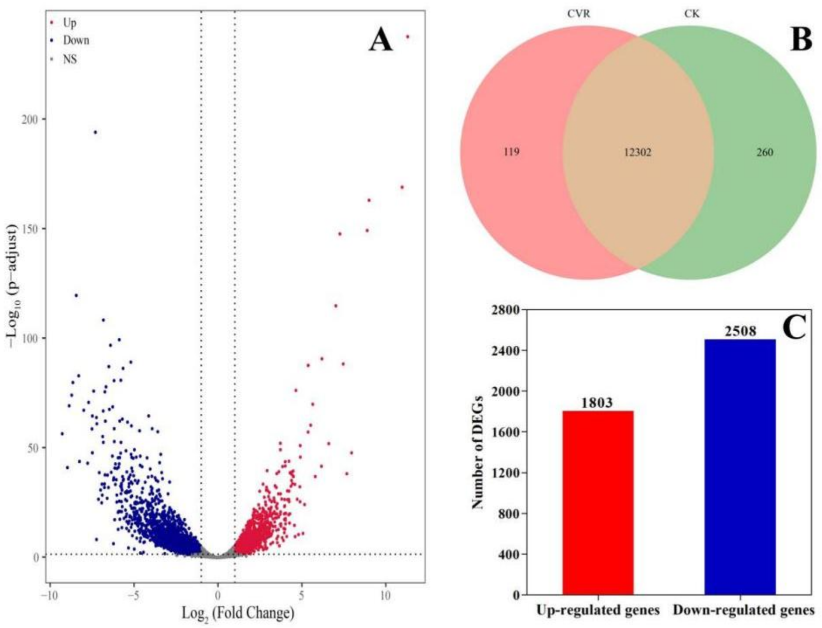

Figure. S2 . Analysis of the KEGG enrichment of differential expressed genes.

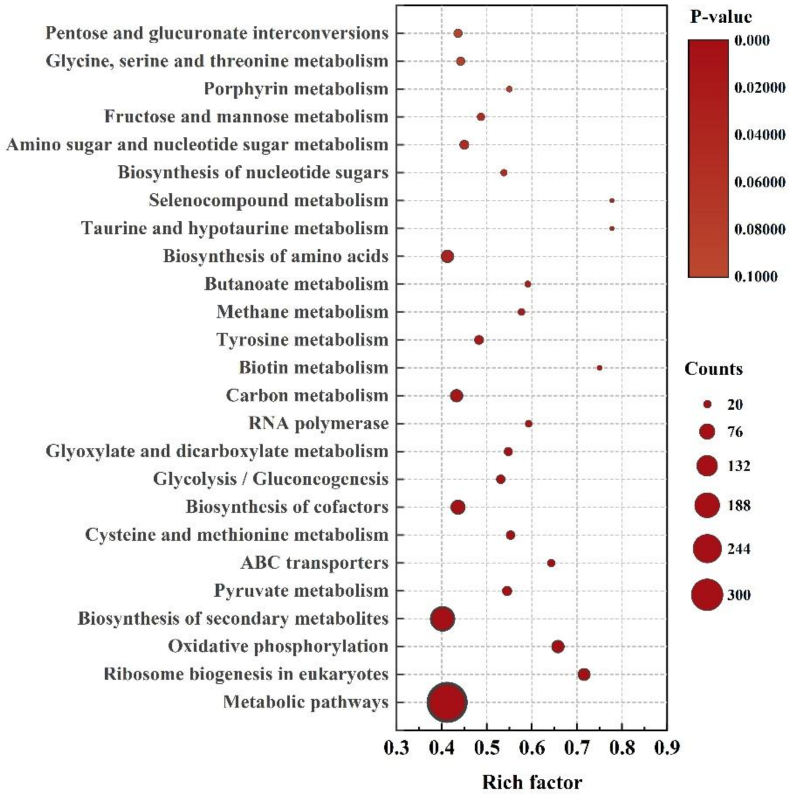

Figure. S3 . Differential gene analysis of RT-qPCR and RNA-Seq. A, Catalase/peroxidase HPI (CC77DRAFT_978519). B, Cytosolic Cu/Zn superoxide dismutase (CC77DRAFT_911584). C, Heme peroxidase (CC77DRAFT_299044). D, Mitochondrial carrier (CC77DRAFT_939153). E, NmrA family protein (CC77DRAFT_580585). F, Putative oxidoreductase (CC77DRAFT_580585). G, Polyketide synthase PksA (CC77DRAFT_1057721). * Represents significant difference according to the student T test ($P < 0.05$) in RT-qPCR. & Represents significant difference according to the student T test ($P < 0.05$) in RNA-Seq.

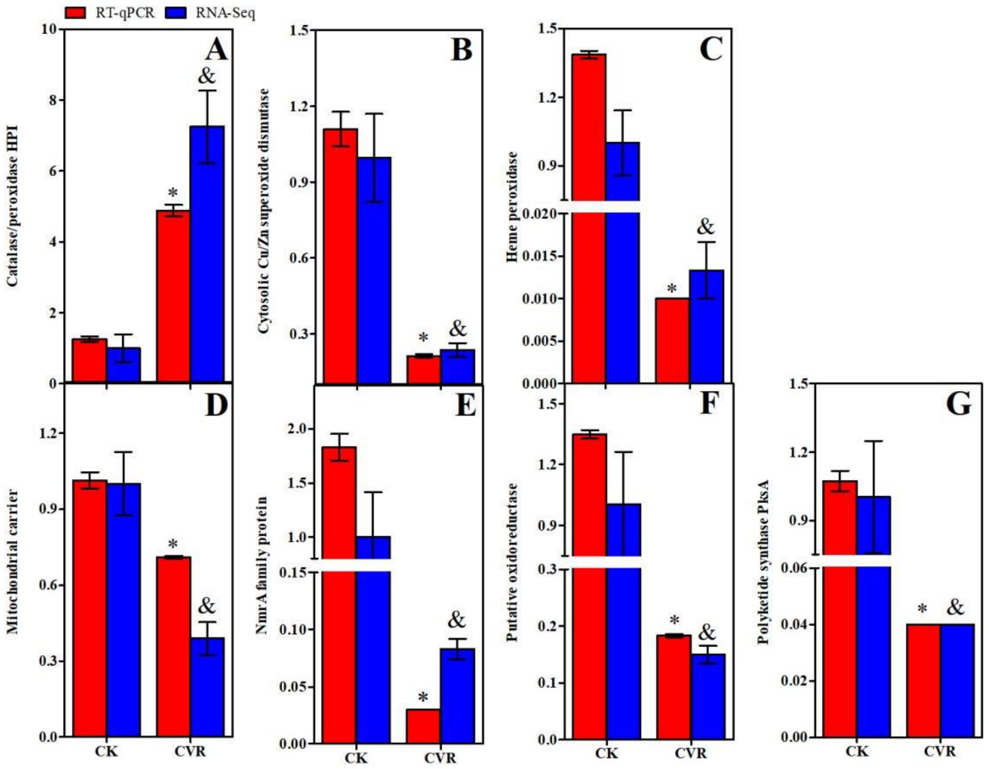

Table S1 . Optimized multiple reaction monitoring (MRM) parameters for AOH, TeA, and AME mycotoxins.

Target Compounds	Inoization Mode	Parent Ion	Qualitative Ion	Retention time (min)	Quantitative Ion	Fragmentation Voltage	Collision Energy (eV)
Altemariol (AOH)	ESI^-	257.0	213.0	4.42	174.0	-100, -98	-32, -48
Tenuazonic acid (TeA)	ESI^-	196.3	138.0	3.51	112.0	-77, -81	-28, -33
Altermariol monomethylether (AME)	ESI^-	270.0	255.7	4.75	228.2	-91, -93	-33, -44

Table S2 . Primers used in RT-qPCR experiment

No.	Gene ID	Gene name	Primer sequence (5’→3’)
1	CC77DRAMN_702782	β-tubulin	F: TCATACTTCGTTGAGTGGAT<br>R: CTGGATGGAGGTGGAGTT
2	CC77DRAFT_978519	Catalase/peroxidase HPI	F: CTGGTGTATCCGTTCCTTTC<br>R: CGAAGAAGTCGTTGGTGAG
3	CC77DRAFT_911584	Cytosolic Cu/Zn superoxide dismutase	F: GGTGACTTGAGCGGAAAG<br>R: GCTGAGATGAACGACGAAG
4	CC77DRAFT_299044	Heme peroxidase	F: GATGCTACACACGCTCTTTG<br>R: AGGACACCCCACCAGTTT
5	CC77DRAFT_1057721	Polyketide synthase PksA	F: TGTATCCGACATCAACAAGG<br>R: TAGCAGGAACCATAGGAAGG
6	CC77DRAFT_939153	Mitochondrial carrier	F: CGGACATTACCCTTGGTTT<br>R: CGAATAGACCCATCTTCCC
7	CC77DRAFT_580585	NmrA family protein	F: ATACACCAAGGAAAGCAAAGA<br>R: GATACGCCTGATGAAACGA
8	CC77DRAFT_1005109	Putative oxidoreductase	F: CGCAACACTTTCCTTTCTG<br>R: GGACCATCATTTTCGCTACT

Table S3 . Summary of the RNA-Seq data in the control (CK) and 0.24 µl/mL carvacrol treated (CVR)

Parameter	CVR1	CVR2	CVR3	CK1	CK2	CK3
Raw Reads	23,889,886	26,238,647	30,512,707	22,732,908	21,456,943	24,928,302
Error Rate (%)	0.03	0.02	0.02	0.02	0.02	0.02
Clean Reads	23,194,375	25,543,829	29,748,442	22,150,628	20,778,802	24,134,725
Clean Reads Rate (%)	97.09	97.35	97.50	97.44	96.84	96.82
Mapped Reads	19,825,787	22,016,352	25,748,670	18,709,456	16,827,570	19,857,640
Mapping Rate (%)	83.23	84.12	84.58	82.56	78.70	79.95
MultiMap Reads	39,441	44,922	54,919	37,264	26,035	33,264
MultiMap Rate (%)	0.17	0.17	0.18	0.16	0.12	0.13
GC Content (%)	54.54	54.67	54.70	54.42	53.88	54.05
Q20 (%)	98.18	98.36	98.48	98.34	98.22	98.30
Q30 (%)	94.93	95.31	95.57	95.32	95.02	95.21
